# Schizophrenia: One Coat of Many Colors

**DOI:** 10.3389/fpsyt.2013.00043

**Published:** 2013-05-27

**Authors:** John Smythies

**Affiliations:** ^1^Center for Brain and Cognition, University of California San DiegoLa Jolla, CA, USA

## Introduction

For a long while the scientific picture of the biochemical origins of schizophrenia presented a large number of apparently quite unconnected isolated findings. Recently however a clearer account has emerged that begins to tie most of these facts together into a single coherent account. In other words, in most cases, a single complex mechanism seems to be involved. The earlier attempts at providing a single hypothesis – for example the transmethylation, dopamine (DA), serotonin, redox, and glutamate hypotheses – are coming to be seen as parts of an interlinked whole.

## Genes

The genetic basis of schizophrenia is complex and does not follow the Mendelian model. Instead it is based on the interactive effect of a large number of abnormal genes each with a single nucleotide polymorphism (SNP) and only a minor impact (Brennand and Gage, 2011). To give a couple of examples out of many to illustrate the complexity of the system: one recent study found 253 SNPs in a population of 92 schizophrenic patients (Chen et al., 2012). Of these SNPs, 138 are known to participate in 100 unique genes that regulate four neurotransmitter pathways – GABA receptor signaling, DA receptor signaling, neuroregulin signaling and glutamate receptor signaling – all processes known to be involved in schizophrenia. Another group (Ayalew et al., 2012), using a different method in a large population of schizophrenics, identified 22 SNPs in genes involved in brain development, myelination, cell adhesion, glutamate receptor signaling, G-protein-coupled signaling, and cAMP-mediated signaling – all process that have to do with interneuronal connectivity. A network of genes involved in the regulation of GAD_67_ shows pronounced changes in expression (i.e., low levels of mRNAs) in schizophrenia (Benes, 2010).

## Epigenetic Processes

Epigenetics refers to mechanisms that control gene expression without changes in DNA sequence and that can be inherited. The mechanisms include DNA methylation and demethylation, acetylation of histones, chromatin-modeling, mRNA splicing/editing, and translation, ribosome biogenesis and, last but not least, micro-RNAs (Millan, 2012).

Many of these epigenetic processes themselves are controlled by synaptic action (Wang and Zhuo, 2012) allowing synaptic control of an extensive range of epigenetic process, that in turn modulate the protein synthesis that is essential for the growth and development of synapses, the dendritic tree and dendritic spines, neural plasticity, long term learning and memory.

In the prefrontal cortex of subjects with schizophrenia, excessive DNA methylation and abnormal histone methylation, at sites of specific genes and promoters, is associated with changes in RNA expression (Akbarian, 2012). The histone acetylation genes and active chromatin remodeling are involved in several stages of long term memory formation, including consolidation, reconsolidation, and extinction. They have also been shown to be abnormal in schizophrenia (Kim et al., 2010).

## DNA Methylation

Recently much interest has focused on the role of transmethylation of nucleic acid bases in schizophrenia. Methylation and demethylation of cytosine bases in DNA represent a prime method of controlling gene expression. In schizophrenia the DNA methylating enzyme Dnmt is over-expressed, which leads to excessive methylation and hence down regulation of the reelin, GAD67, and other genes, resulting in down regulation of the GABAergic INs (Grayson et al., 2005; Grayson and Guidotti, 2013). The activity of Dnmt is regulated by redox factors at several levels (Hitchler and Domann, 2012; Kang et al., 2012).

*S*-adenosylmethionine (the universal methyl group donor derived from methionine) may also play a role in the chemobiology of schizophrenia. It has been known for 50 years that l-methionine exacerbates the symptoms in chronic schizophrenia (Polin et al., 1961; Antun et al., 1971). A similar result is found in mice treated with excess l-methionine, i.e., hypermethylation of the reelin gene and the resulting down regulation of reelin and GAD expression (Dong et al., 2005). The mice also show decreased spine density in frontal cortex. This effect is prevented by valproate, which is an inhibitor of DNA methylation (Dong et al., 2010).

DNA methylation and histone acetylation of the GAD1 gene are heavily influenced by the level of maternal care during the neonatal and preweaning periods (Zhang et al., 2010). These workers showed that pups reared by high grooming/licking (high-LG) dams showed enhanced hippocampal GAD1 mRNA expression, decreased cytosine methylation, and increased histone 3-lysine 9 acetylation (H3K9ac) of the GAD1 promoter. DNA methyltransferase 1 expression was significantly higher in the offspring of low-compared with high-LG mothers. Ketamine effects on PV + neurons are prevented by inhibition of DNA-methylation (Behrens et al., 2010).

## microRNAs

MicroRNAs (miRNA) are short RNAs that control the function of their target messenger RNAs by binding to them by base pairing and so inhibiting further transcription. They are currently the focus of intensive studies in schizophrenia. The most reliable findings come from post mortem studies. Analysis of global miRNA expression in postmortem cortical gray matter from the superior temporal gyrus revealed significant up-regulation of miR-181b expression in schizophrenia (Beveridge et al., 2008). Increased levels of miR-497 have been reported in the prefrontal cortex in schizophrenia (Banigan et al., 2013). Potkin et al. (2010) have identified risk genes with functions related to progenitor cell proliferation, migration, and differentiation, cytoskeleton reorganization, axonal connectivity, and development of forebrain structures. These authors identified in particular several miRNAs as associated with schizophrenia (miRNAs 137, 218, 448).

## Reelin

Reelin is an glycoprotein protease secreted by certain GABAergic interneurons (INs) into the extracellular matrix (Costa et al., 2002). It is involved in corticogenesis, synaptic plasticity, learning, memory, and glutamate NMDA receptor regulation. Expression of reelin is reduced in schizophrenic brain. MRNA levels for a receptor for reelin – very low-density lipoprotein receptor (VLDLR) – are reduced in peripheral blood lymphocytes of schizophrenic patients (Suzuki et al., 2008).

## Neuregulin

Neuregulin (NRG) consists of a family of proteins that plays an essential role in several neurogenerative processes (Bennett, 2011; Ting et al., 2011; Bennett et al., 2012; Fleck et al., 2012). The NRG3 variant rs6584400 is associated with negative symptoms in schizophrenia (Meier et al., 2012).

## Development of the Unifying Hypothesis

The key to this advance was contained in an hypothesis presented by Behrens and Sejnowski (2009) and Wang et al. (2013). They suggested that the initial step in the development of many cases of schizophrenia involved damage to the developing brain by increased levels of the proinflammatory cytokine IL-6 *in utero*, or early in life, derived from such events as a maternal viral infection in cases with a genetic predisposition. IL-6 activates the superoxide-producing enzyme NADPH oxidase (Nox2), and leads to oxidative damage to fast-spiking parvalbumin positive GABAergic INs. A number of receptor proteins important for glutamatergic transmission are regulated by redox sensitive sites (Behrens and Sejnowski, 2009). In adults the effects of superoxide activity are quickly or slowly reversed. However, damage during key period of infancy, especially the second postnatal week is never reversed. This damage results in the later post-pubertal failure to develop the proper circuits in the brain of finely tuned excitation and inhibition between the glutamate and GABA systems. The delay is due to the fact that the GABAergic axons are not fully myelinated until that age. This results in turn in the inability to conduct the complex integration of synchronized oscillations and to cognitive disruption. Fast-spiking INs are highly active and therefore may be more vulnerable to oxidative damage than other cell populations. A related hypothesis was presented by Anderson and Maes (2013).

Further research offers more support for this hypothesis. Nishioka and Arnold. (2004) have reported that levels of 8-OH-2′deoxyguanosine (an oxidative product of DNA) are raised 10-fold in the hippocampus of chronic schizophrenics. IL-6 is actively transferred across the placenta (Zaretsky et al., 2004). Thus maternally derived proinflammatory cytokines could cross the placenta and damage the developing fetal brain. Chronic and acute maternal stress of different types have been associated with increased production of proinflammatory cytokines and decreased levels of anti-inflammatory ones (Leonard and Song, 1998; Maes et al., 1998).

Repetitive administration of the NMDAR antagonist ketamine in animals produces effects akin to the negative symptoms of schizophrenia (Gonzales-Burgos and Lewis, 2013). This may be because the most sensitive NMDARs to ketamine blockade are those located on GABAergic INs. Thus inhibition of these NMDARs will down regulate the GABAergic system (Behrens and Sejnowski, 2009; Anderson and Maes, 2013). This is accompanied with down regulation of the GABA synthesis enzyme GAD67 in PV + INs in rodent brain (Behrens and Sejnowski, 2009) and to an enduring decrease of inhibitory tone in prefrontal cortex (Zhang et al., 2008). In schizophrenia, GABAergic neurons in the prefrontal cortex contain lower levels of glutamic acid decarboxylase 67 (GAD67) mRNA and protein, as well as lower levels of the GABA membrane transporter 1 (GAT1) mRNA. The expression of mRNAs levels for other molecules related to the GABA system – neuropeptide Y, and vasoactive intestinal peptide – are also decreased in postmortem brain, whereas levels of calbindin are increased (Behrens and Sejnowski, 2009).

Behrens and Sejnowski (2009) then review evidence of increased oxidative stress and reduced antioxidant defenses in the brain and body tissues in schizophrenia. They also review a number of instances where this is linked to the increased activity of proinflammatory cytokines. They cite the finding by Smith et al. (2007), using a rodent maternal-infection model, that excess maternal IL-6 so induced results in delayed schizophrenia-like behavior observed in the adult offspring. Further experiments show that prolonged blockade of NMDA-Rs in PV-INs results in an enduring change in AMPA and mGluR5-mediated responses to glutamate (Behrens et al., 2007).

Prenatal exposure of rats to the viral mimic Poly-I:C induced delayed postnatal changes in basal neurotransmitter levels, including reduced DA and glutamate contents in the prefrontal cortex and hippocampus, as well as to deficits in social interaction and anhedonic behavior (Bitanihirwe et al., 2010). Growing evidence from clinical studies with COX-2 inhibitors points to favorable effects of anti-inflammatory therapy in schizophrenia (Müller et al., 2012).

Environmental factors, such as viral infections, inflammation, and obstetrical complications, as well as psychological stress, are closely associated with an increase in oxidative stress (Liu et al., 1996; Lante et al., 2007), and are considered risk factors for schizophrenia (Watson et al., 1984; Brown, 2006, 2011). Social isolation rearing in rats induces behavioral and redox changes akin to schizophrenia (Smythies, 1999; Heidbreder et al., 2000; Andreas et al., 2004). In a study of the effects of 8 weeks social isolation rearing on cortico-striatal redox reactions and social and cognitive behaviors in rats, Möller et al. (2011) found increased superoxide production, decreased levels of the key antioxidant glutathione, and increased lipid peroxidation in several regions, as well induced deficits in prepulse inhibition and social interactive behaviors. Both behavioral and cortico-striatal redox disturbances were corrected by clozapine. A number of antioxidant defense mechanisms have also been shown to be deficient in schizophrenia (Yao et al., 1998, 1999, 2006; Zhang et al., 2009; Yao and Keshavan, 2011).

## Synchronized Gamma Oscillations

Precisely timed interactions between excitatory cortical pyramidal cells and inhibitory PV^+^ GABAergic INs constitute important factors in the synchronization of gamma oscillations (Uhlhaas et al., 2008; Behrens and Sejnowski, 2009). There is evidence that some higher cognitive processes may depend on these (Uhlhaas et al., 2008), particularly at the level of infrastructural neural control (Merker, 2013). Cognitive impairments in schizophrenia may be the result of disturbances in this mechanism (Uhlhaas et al., 2008).

Both excessive and reduced synchronized gamma and other frequency oscillations have been reported in schizophrenia – excessive in the DLPFC by Farzan et al. (2012) and frontal during a working memory task by Barr et al. (2010). Reduced gamma oscillations have been reported by Leicht et al. (2010) and Mulert et al. (2011).

There is also evidence that supranormal gamma synchronization is associated with positive symptoms (“reality distortion”) and subnormal synchronization with negative symptoms (disorganization) (Lee et al., 2003). Clearly this situation is complex and in need of further study.

## Conclusion

The present picture of schizophrenia starts with intrauterine or early postnatal damage to the developing fetal brain by a number of environmental factors, mediated in part by a signaling chain that links cytokine IL-6 and superoxide toxicities in genetically predisposed subjects. These factors focus upon, and disrupt, (1) the function of PV + GABAergic INs, particularly in the prefrontal cortex and (2) DNA methylation and histone epigenetic modifications. These in turn would impair the synchronized oscillations that link PV + INs and P cells in the cortex needed for effective cognition. Excess DNA methylation of the genes for GABAergic relevant molecules would diminish the levels of these molecules in the brain. This central mechanism may activate further signaling chains that involve other brain mechanisms, such as the DA and serotonin systems, lipid peroxidation, etc. Exigencies of space preclude discussion of these earlier hypotheses in any detail. One key point still to be clarified (that has possible therapeutic implications) is to what extent is down regulation of the GABAergic system by oxidative stress due to an irreversible oxidative attack on neural proteins, lipids, and DNA, or to what extent is it the result of potentially reversible DNA methylation and histone modifications.

## References

[B1] Akbarian S. (2012). Epigenetics of schizophrenia. Curr. Top. Behav. Neurosci. 2010, 611–628 10.1007/7854_2010_38 21312415

[B2] Anderson G. Maes M. (2013). Schizophrenia: linking prenatal infection to cytokines, the tryptophan catabolite (TRYCAT) pathway, NMDA receptor hypofunction, neurodevelopment and neuroprogression. Prog. Neuropsychopharmacol. Biol. Psychiatry 42, 5–19 10.1016/j.pnpbp.2012.06.014 22800757

[B3] Andreas L. Feldon J. Ferger B. (2004). Long-term isolation and medial prefrontal cortex: dopaminergic and cholinergic neurotransmission. Pharmacol. Biochem. Behav. 77, 371–379 10.1016/j.pbb.2003.11.011 14751467

[B4] Antun F. Burnett G. B. Cooper A. J. Daly R. J. Smythies J. R. Zealley A. K. (1971). The effects of L-methionine (without MAOI) in schizophrenia. J. Psychiatr. Res. 8, 63 10.1016/0022-3956(71)90009-4 4932991

[B5] Ayalew M. Le-Niculescu H. Levey D. F. Jain N. Changala B. Patel S. D. (2012). Convergent functional genomics of schizophrenia: from comprehensive understanding to genetic risk prediction. Mol. Psychiatry 17, 887–905 10.1038/mp.2012.37 22584867 PMC3427857

[B6] Banigan M. G. Kao P. F. Kozubek J. A. Winslow A. R. Medina J. Costa J. (2013). Differential expression of exosomal microRNAs in prefrontal cortices of schizophrenia and bipolar disorder patients. PLoS ONE 8:e48814 10.1371/journal.pone.0048814 23382797 PMC3559697

[B7] Barr M. S. Farzan F. Tran L. C. Chen R. Fitzgerald P. B. Daskalakis Z. J. (2010). Evidence for excessive frontal evoked gamma oscillatory activity in schizophrenia during working memory. Schizophr. Res. 121, 146–152 10.1016/j.schres.2010.05.023 20598857

[B8] Behrens M. M. Ali S. S. Dao D. N. Lucero J. Shekhtman G. Quick K. L. (2007). Ketamine-induced loss of phenotype of fast-spiking interneurons is mediated by NADPH-oxidase. Science 318, 1645–1647 10.1126/science.1148045 18063801

[B9] Behrens M. M. Hasenstaub A. Sejnowski T. J. (2010). A role for DNA methylation in the NMDA receptor antagonist-mediated loss of phenotype of parvalbumin-positive fast-spiking interneurons. Abstr. Soc. Neurosci. 40, CC2

[B10] Behrens M. M. Sejnowski T. (2009). Does schizophrenia arise from oxidative dysregulation of parvalbumin- interneurons in the developing cortex? Neuropharmacology 57, 193–200 10.1016/j.neuropharm.2009.06.002 19523965 PMC2739086

[B11] Benes F. M. (2010). Regulation of cell cycle and DNA repair in post-mitotic GABA neurons in psychotic disorders. Neuropharmacology 60, 1232–1242 10.1016/j.neuropharm.2010.12.011 21184762

[B12] Bennett M. R. (2011). Schizophrenia: susceptibility genes, dendritic-spine pathology and gray matter loss. Prog. Neurobiol. 95, 275–300 10.1016/j.pneurobio.2011.08.003 21907759

[B13] Bennett M. R. Farnell L. Gibson W. G. (2012). A model of neuregulin control of NMDA receptors on synaptic spines. Bull. Math. Biol. 74, 717–735 10.1007/s11538-011-9706-9 22147103

[B14] Beveridge N. J. Tooney P. A. Carroll A. P. Gardiner E. Bowden N. Scott R. J. (2008). Dysregulation of miRNA 181b in the temporal cortex in schizophrenia. Hum. Mol. Genet. 17, 1156–1168 10.1093/hmg/ddn005 18184693

[B15] Bitanihirwe B. K. Peleg-Raibstein D. Mouttet F. Feldon J. Meyer U. (2010). Late prenatal immune activation in mice leads to behavioral and neurochemical abnormalities relevant to the negative symptoms of schizophrenia. Neuropsychopharmacology 35, 2462–2478 10.1038/npp.2010.129 20736993 PMC3055332

[B16] Brennand K. J. Gage F. H. (2011). Concise review: the promise of human induced pluripotent stem cell-based studies of schizophrenia. Stem Cells 29, 1915–1922 10.1002/stem.762 22009633 PMC3381343

[B17] Brown A. S. (2006). Prenatal infection as a risk factor for schizophrenia. Schizophr. Bull. 32, 200–202 10.1093/schbul/sbj052 16469941 PMC2632220

[B18] Brown A. S. (2011). Exposure to prenatal infection and risk of schizophrenia. Front. Psychiatry 2:63 10.3389/fpsyt.2011.00063 22131978 PMC3222883

[B19] Chen J. Calhoun V. D. Pearlson G. D. Ehrlich S. Turner J. A. Ho B. C. (2012). Multifaceted genomic risk for brain function in schizophrenia. Neuroimage 61, 866–875 10.1016/j.neuroimage.2012.02.064 22440650 PMC3376184

[B20] Costa E. Chen Y. Davis J. Dong E. Noh J. S. Tremolizzo L. (2002). Reelin and schizophrenia: a disease at the interface of the genome and the epigenome. Mol. Interv. 2, 47–57 10.1124/mi.2.1.47 14993361

[B21] Dong E. Agis-Balboa R. C. Simonini M. V. Grayson D. R. Costa E. Guidotti A. (2005). Reelin and glutamic acid decarboxylase67 promoter remodeling in an epigenetic methionine-induced mouse model of schizophrenia. Proc. Natl. Acad. Sci. U.S.A. 102, 12578–12583 10.1073/pnas.0506516102 16113080 PMC1194936

[B22] Dong E. Chen Y. Gavin D. P. Grayson D. R. Guidotti A. (2010). Valproate induces DNA demethylation in nuclear extracts from adult mouse brain. Epigenetics 5, 730–735 10.4161/epi.5.8.13053 20716949

[B23] Farzan F. Barr M. S. Sun Y. Fitzgerald P. B. Daskalakis Z. J. (2012). Transcranial magnetic stimulation on the modulation of gamma oscillations in schizophrenia. Ann. N. Y. Acad. Sci. 1265, 25–35 10.1111/j.1749-6632.2012.06543.x 22823464

[B24] Fleck D. Garratt A. N. Haass C. Willem M. (2012). BACE1 dependent neuregulin processing: review. Curr. Alzheimer Res. 9, 178–183 10.2174/156720512799361637 22455478

[B25] Gonzales-Burgos G. Lewis D. A. (2013). NMDA receptor hypofunction, parvalbumin-positive neurons, and cortical gamma oscillations in schizophrenia. Schizophr. Bull. 38, 950–957 10.1093/schbul/sbs010 PMC3446219 22355184

[B26] Grayson D. R. Guidotti A. (2013). The dynamics of DNA methylation in schizophrenia and related psychiatric disorders. Neuropsychopharmacology 38, 138–166 10.1038/npp.2012.125 22948975 PMC3521968

[B27] Grayson D. R. Jia X. Chen Y. Sharma R. P. Mitchell C. P. Guidotti A. (2005). Reelin promoter hypermethylation in schizophrenia. Proc. Natl. Acad. Sci. U.S.A. 102, 9341–9346 10.1073/pnas.0503736102 15961543 PMC1166626

[B28] Heidbreder C. A. Weiss I. C. Domeney A. M. Pryce C. Homberg J. Hedou J. (2000). Behavioral, neurochemical and endocrinological characterization of the early social isolation syndrome. Neuroscience 100, 749–768 10.1016/S0306-4522(00)00336-5 11036209

[B29] Hitchler M. J. Domann F. E. (2012). Redox regulation of the epigenetic landscape in cancer: a role for metabolic reprogramming in remodeling the epigenome. Free Radic. Biol. Med. 53, 2178–2187 10.1016/j.freeradbiomed.2012.09.028 23022407 PMC3508253

[B30] Kang K. A. Zhang R. Kim G. Y. Bae S. C. Hyun J. W. (2012). Epigenetic changes induced by oxidative stress in colorectal cancer cells: methylation of tumor suppressor RUNX3. Tumour Biol. 33, 403–412 10.1007/s13277-012-0336-0 22274925

[B31] Kim T. Park J. K. Kim H. J. Chung J. H. Kim J. W. (2010). Association of histone deacetylase genes with schizophrenia in Korean population. Psychiatry Res. 178, 266–269 10.1016/j.psychres.2009.05.007 20471694

[B32] Lante F. Meunier J. Guiramand J. Maurice T. Cavalier M. de Jesus Ferreira M. C. (2007). Neurodevelopmental amage after prenatal infection: role of oxidative stress in the fetal brain. Free Radic. Biol. Med. 42, 1231–1245 10.1016/j.freeradbiomed.2007.01.027 17382204

[B33] Lee K.-H. Williams L. Haig A. Gordon E. (2003). “Gamma (40 Hz) phase synchronicity” and symptom dimensions in schizophrenia. Cogn. Neuropsychiatry 8, 57–71 10.1080/713752240 16571550

[B34] Leicht G. Kirsch V. Giegling I. Karch S. Hantschk I. Möller H. J. (2010). Reduced early auditory evoked gamma-band response in patients with schizophrenia. Biol. Psychiatry 67, 224–231 10.1016/j.biopsych.2009.07.033 19765689

[B35] Leonard B. E. Song C. (1998). Stress, depression and the role of cytokines. Adv. Exp. Med. Biol. 461, 251–265 10.1007/978-0-585-37970-8_14 10442177

[B36] Liu J. Wang X. Shigenaga M. K. Yeo H. C. Mori A. Ames B. N. (1996). Immobilization stress causes oxidative damage to lipid, protein, and DNA in the brain of rats. FASEB J. 10, 1532–1538 8940299

[B37] Maes M. Song C. Lin A. De Jongh R. Van Gastel A. Kenis G. (1998). The effects of psychological stress on humans: increased production of pro-inflammatory cytokines and th1-like response in stress-induced anxiety. Cytokine 10, 313–318 10.1006/cyto.1997.0290 9617578

[B38] Meier S. Strohmaier J. Breuer R. Mattheisen M. Degenhardt F. Mühleisen T. W. (2012). Neuregulin 3 is associated with attention deficits in schizophrenia and bipolar disorder. Int. J. Neuropsychopharmacol. 25, 1–8 10.1017/S1461145712000697 22831755

[B39] Merker B. (2013). Cortical gamma oscillations: the functional key is activation, not cognition. Neurosci. Biobehav. Rev. 37, 401–417 10.1016/j.neubiorev.2013.01.013 23333264

[B40] Millan M. J. (2012). An epigenetic framework for neurodevelopmental disorders: from pathogenesis to potential therapy. Neuropharmacology 68, 2–82 10.1016/j.neuropharm.2012.11.015 23246909

[B41] Möller M. Du Preez J. L. Emsley R. Harvey B. H. (2011). Isolation rearing-induced deficits in sensorimotor gating and social interaction in rats arerelated to cortico-striatal oxidative stress, and reversed by sub-chronic clozapine administration. Eur. Neuropsychopharmacol. 21, 471–483 10.1016/j.euroneuro.2010.09.006 20965701

[B42] Mulert C. Kirsch V. Pascual-Marqui R. McCarley R. W. Spencer K. M. (2011). Long-range synchrony of (oscillations and auditory hallucination symptoms in schizophrenia. Int. J. Psychophysiol. 79, 55–63 10.1016/j.ijpsycho.2010.08.004 20713096 PMC3017735

[B43] Müller N. Myint A. M. Krause D. Weidinger E. Schwarz M. J. (2012). Anti-inflammatory treatment in schizophrenia. Prog. Neuropsychopharmacol. Biol. Psychiatry 42, 146–153 10.1016/j.pnpbp.2012.11.008 23178230

[B44] Nishioka N. Arnold S. E. (2004). Evidence for oxidative DNA damage iin the hippocampus of elderly patients with chronic schizophrenia. Am. J. Geriatr. Psychiatry 12, 167–175 10.1097/00019442-200403000-00008 15010346

[B45] Polin W. Cardon P. V. Jr. Kety S. S. (1961). Effects of amino acid feedings in schizophrenic patients treated with iproniazid. Science 133, 104–105 10.1126/science.133.3446.104 13736870

[B46] Potkin S. G. Macciardi F. Guffanti G. Fallon J. H. Wang Q. Turner J. A. (2010). Identifying gene regulatory networks in schizophrenia. Neuroimage 53, 839–847 10.1016/j.neuroimage.2010.06.036 20600988 PMC3055795

[B47] Smith S. E. Li J. Garbett K. Mirnics K. Patterson P. H. (2007). Maternal immune activation alters fetal brain development through interleukin-6. J. Neurosci. 27, 10695–10702 10.1523/JNEUROSCI.0052-07.2007 17913903 PMC2387067

[B48] Smythies J. (1999). Redox mechanisms at the glutamate synapse and their significance: a review. Eur. J. Pharmacol. 370, 1–7 10.1016/S0014-2999(99)00166-1 10323273

[B49] Suzuki K. Nakamura K. Iwata Y. Sekine Y. Kawai M. Sugihara G. (2008). Decreased expression of reelin receptor VLDLR in peripheral lymphocytes of drug-naive schizophrenic patients. Schizophr. Res. 98, 148–156 10.1016/j.schres.2007.12.346 17936586

[B50] Ting A. K. Chen Y. Wen L. Yin D. M. Shen C. Tao Y. (2011). Neuregulin 1 promotes excitatory synapse development and function in GABAergic interneurons. J. Neurosci. 31, 15–25 10.1523/JNEUROSCI.2538-10.2011 21209185 PMC3078582

[B51] Uhlhaas P. J. Haenschel C. Nikolić D. Singer W. (2008). The role of oscillations and synchrony in cortical networks and their putative relevance for the pathophysiology of schizophrenia. Schizophr. Bull. 34, 927–943 10.1093/schbul/sbn062 18562344 PMC2632472

[B52] Wang H. Zhuo M. (2012). Group I metabotropic glutamate receptor-mediated gene transcription and implications for synaptic plasticity and diseases. Front. Pharmacol. 3:189 10.3389/fphar.2012.00189 23125836 PMC3485740

[B53] Wang X. Pinto-Duarte A. Sejnowski T. J. Behrens M. M. (2013). How Nox2-Containing NADPH Oxidase Affects Cortical Circuits in the NMDA Receptor Antagonist Model of Schizophrenia. Antioxid. Redox Signal. 18, 144414–144462 10.1089/ars.2012.4907 PMC3603498 22938164

[B54] Watson C. G. Kucala T. Tilleskjor C. Jacobs L. (1984). Schizophrenic birth seasonality in relation to the incidence of infectious diseases and temperature extremes. Arch. Gen. Psychiatry 41, 85–90 10.1001/archpsyc.1984.01790120089011 6691787

[B55] Yao J. K. Keshavan M. S. (2011). Antioxidants, redox signaling, and pathophysiology in schizophrenia: an integrative view. Antioxid. Redox Signal. 15, 2011–2035 10.1089/ars.2010.3646 21126177 PMC3159108

[B56] Yao J. K. Leonard S. Reddy R. (2006). Altered glutathione redox state in schizophrenia. Dis. Markers 22, 83–93 16410648 10.1155/2006/248387 PMC3850558

[B57] Yao J. K. Reddy R. McElhinny L. G. van Kammen D. P. (1998). Effects of haloperidol on antioxidant defense system enzymes in schizophrenia. J. Psychiatr. Res. 32, 385–391 10.1016/S0022-3956(98)00028-4 9844955

[B58] Yao J. K. Reddy R. van Kammen D. P. (1999). Human plasma glutathione peroxidase and symptom severity in schizophrenia. Biol. Psychiatry 45, 1512–1515 10.1016/S0006-3223(98)00184-X 10356635

[B59] Zaretsky M. V. Alexander J. M. Byrd W. Bawdon R. E. (2004). Transfer of inflammatory cytokines across the placenta. Obstet. Gynecol. 103, 546–550 10.1097/01.AOG.0000114980.40445.83 14990420

[B60] Zhang T. Y. Hellstrom I. C. Bagot R. C. Wen X. Diorio J. Meaney M. J. (2010). Maternal care and DNA methylation of a glutamic acid decarboxylase 1 promoter in rat hippocampus. J. Neurosci. 30, 13130–13137 10.1523/JNEUROSCI.5168-09.2010 20881131 PMC6633524

[B61] Zhang X. Y. Chen da C. Xiu M. H. Wang F. Qi L. Y. Sun H. Q. (2009). The novel oxidative stress marker thioredoxin is increased in first-episode schizophrenic patients. Schizophr. Res. 113, 151–157 10.1016/j.schres.2009.05.016 19540723

[B62] Zhang Y. Behrens M. M. Lisman J. E. (2008). Prolonged exposure to NMDAR antagonist suppress inhibitory synaptic transmission in prefrontal cortex. J. Neurophysiol. 100, 959–965 10.1152/jn.00079.2008 18525022 PMC2525704

